# Post-stroke Quality of Life Index: A quality of life tool for stroke survivors from Sri Lanka

**DOI:** 10.1186/s12955-020-01436-7

**Published:** 2020-07-20

**Authors:** P.K.B. Mahesh, M.W. Gunathunga, S. Jayasinghe, S.M. Arnold, S.N. Liyanage

**Affiliations:** 1 Office of Regional Director of Health Services, Colombo, Sri Lanka; 2 Department of Community Medicine, Faculty of Medicine, University of Colombo, Colombo, Sri Lanka; 3 Department of Clinical Medicine, Faculty of Medicine, University of Colombo, Colombo, Sri Lanka; 4 Colombo East Base Hospital, Colombo, Sri Lanka

**Keywords:** PQOLI, Stroke, Tool development and validation, Quality of life, Sri Lanka

## Abstract

**Background:**

Burden of stroke is rising due to the demographic and epidemiological transitions in Sri Lanka. Assessment of success of stroke-management requires tools to assess the quality of life (QOL) of stroke survivors. Most of currently used QOL tools are developed in high-income countries and may not reflect characteristics relevant to resource-constrained countries. The aim was to develop and validate a new QOL tool for stroke survivors in Sri Lanka.

**Methods:**

The COnsensus-based Standards for the selection of health Measurement Instruments (COSMIN) checklist was referred. A conceptual framework was prepared. Item generation was done reviewing the existing QOL tools, inputs from experts and from stroke survivors. Non-statistical item reduction was done for the 36 generated items with modified-Delphi technique. Retained 21 items were included in the draft tool. A cross sectional study was done with 180 stroke survivors. Exploratory Factor Analysis was done and identified factors were subjected to varimax rotation. Further construct validity was tested with 6 a-priori hypothesis using already validated tools (SF-36, EQ-5D-3 L) and a formed construct. Internal consistency reliability was assessed with Cronbach alpha.

**Results:**

Four factors identified with principal-component-analysis explained 72.02% of the total variance. All 21 items loaded with a level > 0.4. The developed tool was named as the Post-stroke QOL Index (PQOLI). Four domains were named as “physical and social function”, “environment”, “financial-independence” and “pain and emotional-wellbeing”. Four domain scores of PQOLI correlated as expected with the SF-36, EQ-5D Index and EQ-5D-VAS scores. Higher domain scores were obtained for ambulatory-group than the hospitalized-group. Higher scores for financial-independence domain were obtained for the group without financial-instability. Five a-priori hypothesis were completely proven to be true. Cronbach-alpha level ranged from 0.682 to 0.906 for the four domains.

**Conclusions:**

There is first evidence for sufficient construct validity of the PQOLI as a valid QOL tool for measuring the QOL of stroke survivors with satisfactory internal consistency reliability.

## Introduction

Stroke is a rapid vascular event with symptoms lasting 24 h or longer [1]. Stroke is regarded as a major cause of disability anywhere in the world [2]. Its burden has been relatively worsening in low- and middle-income-countries (LMICs) compared to high-income countries (HICs) in relation to its incidence as well as social and economic impacts [3]. As an example when a decrease of 42% of stroke incidence is observed in HICs, an increase over 100% is observed in LMICs over the last four decades [3]. Sri Lanka was recently upgraded as a Upper-Middle Income Country after being a LMIC with comparatively satisfactory health parameters [4, 5]. Prevalence of stroke in urban Sri Lanka was estimated to be 10.4 per 1000 (95% CI = 6.3 to 14.5) with a male to female ratio of 2:1 [6]. Relatively higher incidence of stroke among the young also been noted here [7]. With the demographic and the epidemiological transitions, as in many other LMICs, stroke burden is expected to rise further in future.

The World Health Organization (WHO) defines Quality of Life (QOL) as “the individual’s perception of his/her position in life in the context of the culture and value systems in which they live and in relation to their goals” [8]. It is a composite concept affected by many facets [8]. In a patient with a disease condition, the QOL may reflect the success of management of that particular disease, as the patient perceives [9]. Disabilities imposed by stroke would directly or indirectly influence the physical as well as psychological QOL components as perceived by the patients. Hence the QOL of stroke survivors is an extremely important factor, for research, to assess progress and to target services for stroke survivors [10–12].

Since QOL is a patient-reported outcome, there is no agreement on exactly which domains are to be captured within its scope [13]. Hence, though there are many available QOL tools, there is much variability among their domains [14, 15]. Environmental influences (such as user-friendliness and safety of household items) and economic influences (such as financial security), do affect the “self-perceived position in life”, especially in disability-related conditions like stroke. Many QOL tools do not capture these, even though these are proved to impose an impact on overall QOL [16–22]. It has been encouraged to design QOL instruments specially including domains like economic costs and burden to the family members [23]. Furthermore, epidemiological characteristics of stroke are prone to vary even within LMICs [24, 25]. The living contexts (i.e. standards of household environment and items) and the expectations of the people (i.e. due to the differences in salary scales and in social insurance systems) are different between the HICs and LMICs. Stroke-related literature is scarce in relation to LMICs. Hence most of the evidence on stroke rehabilitation which come from Western settings which might not be applicable for lower-middle income settings [26–30]. The development of new QOL tools for stroke survivors would enable more context-related QOL measurements [31].

Out of the many health-related QOL tools available, Short-Form-36 (SF-36) and European Quality of Life 5 dimensional (EQ-5D) tool are two commonly used generic tools [9, 32–34]. SF-36 includes 08 scales and has been used to assess QOL in stroke survivors following hospital discharge [35–39]. It has been validated for several disease conditions including stroke within Sri Lanka [37, 40]. EQ-5D tool consists of an EQ-5D descriptive system as well as an EQ-Visual-Analogue-Scale (VAS). The former system include five dimensions and the latter records the respondent’s self-rated health on a VAS [41]. It too has been validated within Sri Lanka [42, 43]. SF-36 covers a period of 28 days previous to the date of data collection and the EQ-5D captures the QOL related to the time of data collection.

Validity, reliability and responsiveness are three properties that must be evaluated in relation to a newly developed measurement tool [44, 45]. Validity measures whether the tool “actually measures what it is expected to measure” [46]. In the absence of a gold standard test for QOL, the validity based on data is assessed by the construct validity [47]. Reliability refers to a measure of inherent amount of error of any measurement [48]. It includes the assessment of homogeneity of each of the items measured by Cronbach Alpha and reproducibility (measured with test-retest method etc.) [48, 49]. Responsiveness is defined as the ability to detect a clinically important change [50, 51].

Sri Lanka is trying to expand stroke-related services in general including that of acute care, rehabilitation and community-based care [52]. Availability of context-specific QOL tools would greatly help in assessing the success of management of stroke survivors in this context. The aim of the study was to develop and validate a comprehensive quality of life tool for stroke survivors at post-stroke 1-month in Sri Lanka.

## Methods

### Conceptual framework and item generation

**Fig. 1:**
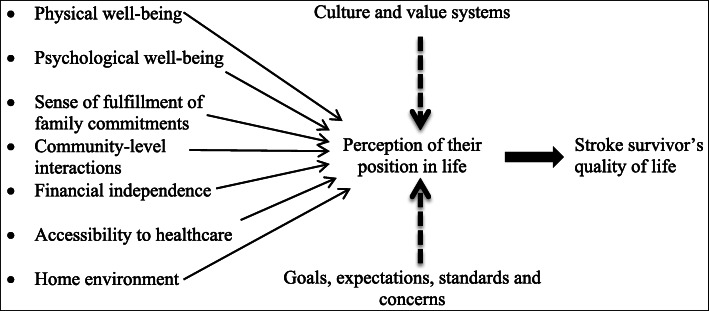
Conceptual framework

The COnsensus-based Standards for the selection of health Measurement Instruments (COSMIN) checklist was referred [44, 45]. The literature was searched using electronic databases (PUBMED and MENDELEY) with relevant key words (Supplementary file-I). General and specific tools that measure QOL of the stroke survivors were noted. Having studied the variables/domain of the identified tools, inputs from key informants and clients (three survivors of stroke and two caregivers), a conceptual framework was developed (Fig. 1). The identified domains were within the scope of the interpretation of QOL by the WHO [8].

Using the conceptual framework, items were generated and listed by reviewing the items of identified tools, getting additional inputs from expert key-informants and clients mentioned above. Items were expected to cover the quality of life of a period of 7 days of the stroke survivors’ life, from the time of interview.

In all the above processes inputs were taken from a panel of seven key informants who were experts related to the stroke care. The panel included two consultants in neurology in the premier tertiary hospital of Sri Lanka, one consultant in clinical medicine and anesthesiology from the same setting. Furthermore it included a professor in community medicine and a lecturer in sociology in a premier university as well as a general practitioner.

### Non-statistical item reduction and drafting the questionnaire

Similar items were combined. This draft list was sent to the eight experts (i.e. seven above mentioned panel members and another nominated by them) above with modified Delphi technique through emails. They were asked to rate each item with a five-point scale based on the relevance of the question to the local setting (i.e. from least relevant to extremely relevant) and to send back their ratings through email. The items whose average scores were within the first quartile were omitted.

The stems were drafted for these items in par with both an interviewer-administered and a self-administered questionnaire. It is highlighted in the literature, that a tool, being able to be used as both self and interviewer administered, is important in relation to stroke [53]. The concepts described by Streiner and Norman (2008), like conceptual equivalence, item equivalence, semantic equivalence and translation procedures were considered in drafting the stems [54]. The questions were originally formed in English and then translated to Sinhalese. The forward-and- backward-translation method was used adhering to the recommended measures [55].

Five response categories were used for each stem. The response categories ranged from “Always” to “Not at all”. A scoring system from 1 to 5 with 1 representing the worst QOL and 5 representing the best was developed. Stems representing negative phenomena were reverse coded. The items thus generated, were given to the panel of experts to allocate a mark out of 10 per each item in relation to the wording and clarity.

### Data collection and factor analysis

A cross sectional study was carried out in two settings to explore the factor structure of the draft instrument. The rehabilitation unit of the Colombo North Teaching Hospital was one setting. Residential rehabilitation services for the stroke survivors are available here. The other setting was the neurology clinics of the National Hospital of Sri Lanka where the ambulatory care is given for patients after the discharge from the hospital. Stroke survivors who were within 28 days to 32 days following the acute phase of their management were selected to the study leaving a margin of 2 days from either side of the intended period of 1 month. In order to obtain reliable estimates, it has been mentioned that a minimum subject to item ratio of 1: 5 is needed for exploratory factor analysis [56]. Since the interviewer-administered questionnaire contained 21 retained questions it was decided to have 7.5 times of the number of variables making the sample size as 157 considering the feasibility of data collection. With an assumed response rate of 90%, the sample size at the data collection stage was decided to be 180. The participants who were managed at study settings from 1st of December 2014 were eligible to be included in the study.

### Exploratory factor analysis

Data was entered in to a Statistical Package of Social Sciences (version 17) datasheet. Factorability was assessed using the Kaiser-Meyer-Olkin (KMO) measure of sample adequacy, Bartlett’s test of sphericity and anti-image correlations [57]. Exploratory Factor Analysis with Principal Component Analysis was done. We retained factors whose Eigenvalues were greater than 1. Having studied the scree plots, the selected factors were subjected to varimax rotation. The subsequent factor loadings were examined.

The draft instrument was pre-tested among 10 stroke survivors. Following the administration of the questionnaire, the investigators had a brief interview with them on the wording of items of the questionnaire. Based on the responses, the final adjustments were done in relation to the clarity of wording.

### Further assessment of validity and reliability of the developed tool

The three aspects of the judgmental validity; face, content, consensual (i.e. agreement between experts) were assessed with the inputs of the panel of experts [47]. The data-based further construct-validity was assessed using the findings of SF-36, EQ-5D and the questions on financial burden.

Six a-priori hypotheses were used.

1. The domain scores of the developed tool will have statistically significant positive correlations with all domains of SF-36
2. The domain scores of the developed tool will have statistically significant positive correlations with EQ-5D index score
3. The domain scores of the developed tool will have statistically significant positive correlations with EQ-5D VAS score
4. The domain scores of the developed tool will correlate with more-related domains with a higher strength of association than the scores of other domains.

The a-priori hypotheses from no. 1 to no. 4 were evaluated by testing the construct validity with the findings of SF-36 and EQ-5D-3-Level tools.

5. The domain scores of the developed tool will be statistically significantly higher for the ambulatory group than the hospitalized group. The hypothesis no.05 tested the ability of the tool to discriminate between the two ends of the severity spectrum, as another aspect of construct validity. For this, the domain scores of the institutionalized participants and the ambulatory participants were compared.
6. There will be a statistically significant difference of the financial domain scores between the groups with financial burden at family level and those who are not. The sixth was evaluated by a set of four judgmentally-validated questions composed by reviewing the literature on financial burden [58]. The questions were; whether the participant had to apply for a loan, whether the participant had to sell a property, whether the participant had a reduction of income and whether the participant had to restrict the expenses for other usual matters due to the impact of the disease condition. The presence of any of these was noted as presence of financial burden at family-level.

This procedure of adopting a-priori hypotheses was similar to the methodology adapted in many other global validation studies [59–62]. Satisfactory confirmation of two thirds of the hypotheses at least was considered as necessary for sufficient construct validity.

Internal consistency reliability was assessed by calculating the Cronbach alpha coefficient. Internal consistency estimates of a magnitude of 0.70 or greater was considered as satisfactory [63]. Level of significance was considered as 5%. Ethical clearance was obtained from the Ethics Review Committee of the Medical Research Institute of Sri Lanka (Reference 60/2014). The procedures followed were in accordance with institutional guidelines of the study settings. Informed written consent was obtained from the participants.

## Results

The domains of QOL that have been included in the tools found in literature search have been listed in Supplementary file 2 [10, 53, 64–68].

**Fig. 2:**
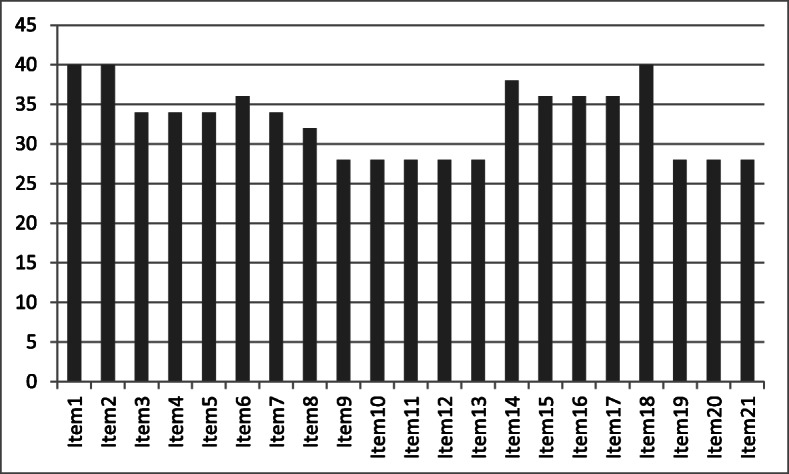
Cumulative scores given by the expert panel for the selected items (x axis corresponds each item and the y axis shows the cumulative score)

After combining the related items, the initial list included 36 items. The mean and median cumulative scores given by the expert panel through the modified-Delphi technique for the initial item list was 22 and 28 respectively. The upper margin of the first quartile was 7. The number of items which scored more than the upper first quartile was 21. Figure 2 shows the distribution of the cumulative scores given by the experts. No outliers were detected for the scores on clarity and wording.

It took in average 20 min for an interview with a participant. The median (Inter Quartile Range) age of the sample was 56 (46–65). The total respondents were 180 with the male to female ratio of the participants being approximately 2.8 to 1 (73 to 27%). The proportions of missing values ranged from 1.1 to 11.1%. The KMO measure of sampling was 0.876 and the Barlett test of sphericity was significant (*p* < 0.001). The diagonal anti-image correlation ranged from 0.611 to 0.943. Following the factor analysis, four factors were detected with an eigenvalue more than 1. Out of those 04, the maximum eigenvalue was 10.051 and the least value was 1.182. Out of the total variance, 72.02% was explained by the cumulative variance of these four factors. All the 21 items loaded with a level > 0.4 into the selected four components. Hence no further statistical item reduction was done.

The factor structure analysis of the 21 items is mentioned in Table 1.

**Table 1:** Factor structure analysis of the tool (each item corresponds to the respective question number of the PQOLI- as shown in Table 3)

	Factor 1	Factor 2	Factor 3	Factor 4
Item1	**.858**	.237	.191	.163
Item2	**.886**	.228	.175	.103
Item3	**.852**	.187	.221	.134
Item4	**.540**	.077	.167	.341
Item5	**.838**	.102	.185	.147
Item6	**.859**	.008	.043	.101
Item7	**.750**	.234	.302	.048
Item8	**.613**	.448	.453	.065
Item9	**.590**	.526	.291	.061
Item10	**.491**	.508	.428	−.176
Item11	.482	.245	**.642**	.087
Item12	.219	.151	**.781**	−.014
Item13	.167	.084	**.854**	.049
Item14	.512	.231	−.002	**.508**
Item15	.095	.222	.**691**	.303
Item16	.471	.254	.308	**.476**
Item17	.138	−.006	.088	**.863**
Item18	.167	**.752**	.273	.069
Item19	.096	**.863**	−.016	−.019
Item20	.142	**.859**	.172	.127
Item21	.211	**.784**	.179	.135

Considering the factor loadings and the correlations of the factors, items were assigned into the respective factors. The complex variables were retained in the best factor as suggested by the Cronbach alpha values with “items removed”. The Table 2 summarizes the correspondence of the retained items to the individual domains.

**Table 2:** Correspondence of the retained items to the four domains

	Domain I	Domain II	Domain III	Domain IV
Given name	Physical and social functioning	Environment	Financial independence	Pain and emotional well-being
Included no. of items	10	4	4	3
Item numbers	Item 1	Item 18	Item 11	Item 14
	Item 2	Item 19	Item 12	Item 16
	Item 3	Item 20	Item 13	Item 17
	Item 4	Item 21	Item 15	
	Item 5			
	Item 6			
	Item 7			
	Item 8			
	Item 9			
	Item 10			

The developed tool was named as “Post-stroke Quality Of Life Index for strokes survivors” (PQOLI) (Table 3).

**Table 3:** The PQOLI tool

Questions are covering the last 7 days	Best responses	
1. My illness/s have a negative effect on my ability to walk and move around	Not at all	
	Rarely	
	Occasionally	
	Frequently	
	Always	
2. My illness/s have a negative effect on the ability i used to have to look after myself	Not at all	
	Rarely	
	Occasionally	
	Frequently	
	Always	
3. My illness/s have a negative effect on my ability to select foods, cook, serve or to eat that I used to have	Not at all	
	Rarely	
	Occasionally	
	Frequently	
	Always	
4. My illness/s have a negative effect in having a sound sleep	Not at all	
	Rarely	
	Occasionally	
	Frequently	
	Always	
5. My illness/s have a negative effect in my ability to have sexual activities that I used to have	Not at all	
	Rarely	
	Occasionally	
	Frequently	
	Always	
6. My illness/s cause discomfort in proper toilet practices	Not at all	
	Rarely	
	Occasionally	
	Frequently	
	Always	
7. My illness/s have a negative effect on my ability to communicate with others	Not at all	
	Rarely	
	Occasionally	
	Frequently	
	Always	
8. My illness has restricted me performing previous roles I played in my family	Not at all	
	Rarely	
	Occasionally	
	Frequently	
	Always	
9. The society assumes that I am not capable of performing social activities that I used to do due to my illness	Not at all	
	Rarely	
	Occasionally	
	Frequently	
	Always	
10. My illness has restricted me performing previous recreational activities I used to have	Not at all	
	Rarely	
	Occasionally	
	Frequently	
	Always	
11. My income-generation activities are restricted by the illness	Not at all	
	Rarely	
	Occasionally	
	Frequently	
	Always	
12. I am worried about the restrictions on life by having to be on regular attention (medications, attending clinics, getting investigations)	Not at all	
	Rarely	
	Occasionally	
	Frequently	
	Always	
13. I am worried about the negative effect of my illness on my/my family’s financial stability	Not at all	
	Rarely	
	Occasionally	
	Frequently	
	Always	
14. I am generally satisfied about the way I live in spite of my illnesses	Not at all	
	Rarely	
	Occasionally	
	Frequently	
	Always	
15. I worry about my or my family’s future as a result of my illness/s	Not at all	
	Rarely	
	Occasionally	
	Frequently	
	Always	
16. Due to the illness I suffer from pain	Not at all	
	Rarely	
	Occasionally	
	Frequently	
	Always	
17. My health condition is getting worse with time	Not at all	
	Rarely	
	Occasionally	
	Frequently	
	Always	
18. The equipment of my house (bed, chairs, equipment, toilet accessories) is not user-friendly considering my health condition	Not at all	
	Rarely	
	Occasionally	
	Frequently	
	Always	
19. The living environment of my house (floor, stairs, space) is not user-friendly considering my health condition	Not at all	
	Rarely	
	Occasionally	
	Frequently	
	Always	
20. I am worried about the transport in accessing health care	Not at all	
	Rarely	
	Occasionally	
	Frequently	
	Always	
21. I have no restriction or problem in getting the medical care (medical advice, drugs, investigations)	Not at all	
	Rarely	
	Occasionally	
	Frequently	
	Always	

The measures of location and measures of dispersion were obtained as shown in Table 4. All domain scores have a potential range from 0 to 100.

**Table 4:** Characteristics of the domain scores of the PQOLI tool

Domain	Mean	Standard deviation	Median	Inter-Quartile Range
I score	56.17	22.35	53.00	40.00–72.00
II score	58.15	20.23	60.00	40.00–70.00
III score	44.25	16.74	40.00	33.75–55.00
IV score	66.93	20.69	66.67	53.33–86.67

Table 5 shows the spearman-correlation of the domain scores with the scores of SF-36 and the EQ-5D tools.

**Table 5:** Correlation of domain scores of PQOLI with SF-36 and EQ-5D

	Domain I Correlation (r_s_)^a^ Significance	Domain II Correlation (r_s_)^a^ Significance	Domain III Correlation (r_s_)^a^ Significance	Domain IV Correlation (r_s_)^a^ Significance
General Health	0.352 *p* < 0.001^b^	0.249 *p* < 0.001^b^	0.195 *p* < 0.001^b^	0.296 *p* < 0.001^b^
Physical function	0.792 *p* < 0.001^b^	0.416 *p* < 0.001^b^	0.476 *p* < 0.001^b^	0.667 *p* < 0.001^b^
Pain	0.761 *p* < 0.001^b^	0.405 *p* < 0.001^b^	0.485 *p* < 0.001^b^	0.612 *p* < 0.001^b^
RL- physical	0.581 *p* < 0.001^b^	0.524 *p* < 0.001^b^	0.468 *p* < 0.001^b^	0.372 *p* < 0.001^b^
RL- emotional	0.502 *p* < 0.001^b^	0.343 *p* < 0.001^b^	0.481 *p* < 0.001^b^	0.382 *p* < 0.001^b^
Vitality	0.403 *p* < 0.001^b^	0.388 *p* < 0.001^b^	0.305 *p* < 0.001^b^	0.276 *p* < 0.001^b^
Social functioning	0.759 *p* < 0.001^b^	0.408 *p* < 0.001^b^	0.474 *p* < 0.001^b^	0.570 *p* < 0.001^b^
Mental Health	0.343 *p* < 0.001^b^	0.340 *p* < 0.001^b^	0.476 *p* < 0.001^b^	0.339 *p* < 0.001^b^
EQ-5D index	0.845 *p* < 0.001^b^	0.489 *p* < 0.001^b^	0.563 *p* < 0.001^b^	0.812 *p* < 0.001^b^
EQ-5D VAS	0.745 *p* < 0.001^b^	0.381 *p* < 0.001^b^	0.476 *p* < 0.001^b^	0.667 *p* < 0.001^b^

^a^Spearman correlation-coefficient ^b^significant association

RL-physical- Role-Limitations-physical

RL-Emotional- Role-Limitations-emotional

EQ-5D-Index- Index score of the Euro-QOL-5D tool

EQ-5D-VAS- Visual Analogue Scale score of Euro-QOL-5D tool

All domains showed statistically significant positive correlations with scores of SF-36 and EQ-5D-3 L. The EQ-5D-3 L scores had relatively higher strength of associations for the Domains I and IV. In general, for the domain scores with more similar constructs of the SF-36, the associations were with relatively higher strengths.

The ability to discriminate between the two ends of the severity spectrum of a disease entity is mentioned in Table 6. It shows that the QOL was significantly higher in the ambulatory group than the institutionalized group.

**Table 6:** Discrimination ability of PQOLI

	Hospital group (*n* = 99)	Ambulatory group (*n* = 81)	Significance of differenc^a^
Domain I median (IQR)	40.00 (30.45–52.73)	72.72 (59.09–90.00)	*p* < 0.001^b^
Domain II median (IQR)	50.00 (40.00–60.00)	60.00 (50.00–85.00)	*p* < 0.001^b^
Domain III median (IQR)	35.00 (30.00–40.00)	50.00 (40.00–60.00)	*p* < 0.001^b^
Domain IV median (IQR)	60.00 (40.00–80.00)	90.00 (80.00–90.00)	*p* < 0.001^b^

^a^Mann-Whitney U test ^b^Significant association

*IQR* Inter-Quartile-Range

In the study sample, 27%(*n* = 49) had to apply for a loan, 30%(*n* = 54) had to sell a property, 67%(*n* = 120) had a potential income loss and 67% (n = 120) had to restrict other expenses, due to the illness. Approximately 73% (*n* = 133) had a financial burden. Higher values for the financial-domain-score were obtained by those who did not experience a financial burden. The median (IQR) financial-domain-scores of PQOLI for the group with financial burden was 35.00 (25.0–50.0). The relevant scores of the group without financial burden was 40.00 (35.0–55.0). There was a statistically significant difference between these two groups (*p* = 0.012).

Cronbach alpha level for the domains I,II,II and IV were respectively 0.906, 0.880, 0.803 and 0.682.

## Discussion

The PQOLI was developed upon context specific evidence of the study setting. It includes 21 items under four domains of physical/social, pain/emotional, financial stability and environmental domains. Hence it addresses many of the deficiencies of utilizing current QOL measures within the resource-constrained settings [26–29, 31].

Many of the recommended methodological aspects in the development of QOL tools were adhered in the development of the PQOLI [44]. As recommended in global literature, patients too were involved in several components of the study including the phase of item generation [33, 69]. The factorability assessments done by KMO measure, Barlett test, item communalities and anti-correlation images were satisfactory [57, 70, 71]. The KMO measure was more than 0.8 and hence can be classified as “meritorious” [57]. The significant Barlett test suggests that the “correlation matrix was significantly different from the identity matrix and therefore factorable” [57].

Exploratory Factor Analysis is better suited when the domain structure is not previously known as in this study [70]. Principal Component Analysis assumes the continuous nature of the variables and it was ensured by the presence of six response categories for each item [71]. It was performed following that with the intention of having a smaller number of variables that explain the most variation in the original set. Even though the orthogonal varimax rotation is commonly done, as in the present study, oblique techniques have been recommended in some literature [69, 72]. A minimal eigenvalue of 1 is traditionally used in defining the factors as in the present study [61]. All the items had a loading value more than the traditional cut-off of 0.32 [69]. The complex factors with cross-loadings were dealt with internal consistency analysis [69, 72, 73] The Cronbach’s alpha values with “items removed” suggest that the allocation of cross loadings were accurate [74].

PQOLI is a disease-specific QOL tool in contrast to generic tools [75]. Disease-specific tools are considered better than generic tools for being more sensitive in picking the changes of stroke patients [33]. The present tool was developed to cover a recall period of seven days in contrast to the four-weeks recall period of SF-36 and momentary capturing of QOL by EQ-5D [35, 41]. The validity of PQOLI was assessed at one month from the end of acute-management of the stroke. In literature post-stroke QOL has been measured from one month up to five years [76]. The seven-day recall period and utilization at one-month were decided in considering the rehabilitation procedure of the study settings and by considering the quality of patient-responses in the pre-testing period.

Following the factor analysis, financial and environmental components were identified as two domains in the PQOLI. This is an example of the recent global recommendation that domain structures for QOL tools for LMICs should be context specific [31]. Furthermore this proves the necessity of incorporating economic and environmental domains in QOL measurements [16–22]. In many LMICs like Sri Lanka, social-insurance systems are not found. The services not free-of-charge, may have to be achieved by out-of-pocket expenditure creating a potential financial burden. These may be the reasons for getting a financial-independence domain following factor analysis. Domain II proves that the stroke-survivor-friendliness of the lining environment and the accessibility to healthcare too are important in determining the quality of life.

The pattern of correlations, provides evidence that the a-priori hypotheses no.1 to no.4 are fulfilled. This reflects that the PQOLI was based on a reflective model in addition to the assessment with the “thought test” as recommended in the COSMIN checklist [44]. All the items can be assumed to be changed when the underlying construct changes [44]. In evaluating the construct validity by the correlations, significance, direction and effect size are generally considered [44]. However due to the “complexities of the constructs”, the strength of associations cannot be expected to be interpreted in the exact way as in bivariate correlations. Since then, for the a priori-hypotheses 1 to 3, the significance and the directions were included while the explorations of strengths were focused in hypothesis no.4 [77, 78].

The hypothesis no.4 was fulfilled in relation to domain I, but was not to a great extent for domain IV. The domain structure in the PQOLI and the SF-36 are not the same. As an example, the pain domain which is a separate entity in the SF-36, has been included in the domain IV of the PQOLI together with items related to emotional-health. This difference of the item-structure might be one reason for PQOLI domain IV not having a greater strength with the mental-health, vitality and role-limitation-emotional domains of SF-36. However, the strength of associations were still of “acceptable and of medium strength” [77–79].

As expected the domain scores of PQOLI were significantly higher for the ambulatory group than the hospitalized group. This highlights its ability to discriminate between the severities of a spectrum. Institutionalized patients are with a higher severity of illness than the patients in the ambulatory group whom have been discharged from the in-ward care. This fulfills the a-prori hypothesis no.5. Hypothesis no.6 tested the ability of the Domain III to discriminate between groups with differences in the level of financial-independence. A lower financial-independence in the group with “financial-burden” would have lowered their subjective perception of the position of living, thus lowering the QOL. This has been accurately captured by the Domain III of PQOLI. In a summary, five of the six a-priori hypotheses have been completely fulfilled and one has been partially fulfilled. Since the alpha values for domains I,II and III were more than 0.8 and for domain IV being closer to 0.7, it can be classified as demonstrating satisfactory internal consistency [44, 70, 80].

Several limitations can be mentioned in this process. One is that the PQOLI provides only numerical outcome-scores for the domains without providing a categorical outcome as “satisfactory” and “not-satisfactory”. This is acceptable as establishing a cut-off needs research in larger scale and it would be a future extension of this. Secondly the PQOLI provides 4 domain scores rather than an amalgamated score. Such a score would need weighing of the domains and that too would be done as a future extension of this research which would be done in a larger scale. The tool underwent exploratory factor analysis and it was not followed by confirmatory factor analysis in another study sample. Hence it is advisable to conduct confirmatory-factor analysis when this is used in another setting.

The reproducibility was not assessed for PQOLI and only the internal consistency reliability was assessed [80–82]. Similarly the responsiveness of the instrument was also not assessed [50, 51]. These reflect further directions on the future research o PQOLI. There were limited descriptive data on the characteristics of the study sample. Though this was done to minimize the time of data collection, it is another limitation of the study. However, in order to minimize any selection bias, participants with a history of physical or mental conditions that would affect the quality of life were excluded. Furthermore, the study settings were with free healthcare and were without any restriction of access [83].

## Conclusions

PQOLI included 21 items which are categorized under 04 domains in its development. There is first evidence for sufficient construct validity of the PQOLI as a valid QOL tool for measuring the QOL of stroke survivors with satisfactory internal consistency reliability, when assessed using already validated QOL tools and with the “known group comparison method”. Five out of the six a-prori hypotheses were completely fulfilled in testing it for validity. Its internal consistency reliability was reflected to be satisfactory. PQOLI can be used for the assessment of QOL after 1 month from the end of acute-phase of management of the stroke survivors, following further explorations.

## Supplementary information

**Additional file 1.** PubMed search strategy. Mendeley search terms.

**Additional file 2.** Domain analyses of commonly used QOL tools for stroke patients.

## Data Availability

The datasets used and/or analyzed during the current study are available from the corresponding author on reasonable request.

## Abbreviations

QOL: Quality of Life
PQOLI: Post-stroke Quality Of Life Index
COSMIN: COnsensus-based Standards for the selection of health Measurement Instruments
WHO: World Health Organization
SF-36: Short Form 36
EQ-5D-3 L: EuroQOL 5-Dimentional 3-level questionnaire
EQ-VAS: EuroQOL Visual Analogue Scale
LHS: London Handicap Scale
NHP: Nottingham Health Profile
SIP: Sickness Impact Profile
KMO: Kaiser-Meyer-Olkin
