# Supplementary material for: Post-stroke Quality of Life Index: A quality of life tool for stroke survivors from Sri Lanka
Source: Health Qual Life Outcomes. 2020 Jul 20;18:239. doi: 10.1186/s12955-020-01436-7 (PMC7370468; doi:10.1186/s12955-020-01436-7)
Supplement: Supplementary file 1 — Additional file 1. PubMed search strategy. Mendeley search terms. [file 12955_2020_1436_MOESM1_ESM.docx]

**Supplementary file 1**

**PubMed search strategy:**

PubMed- (((((((((("Stroke"[Mesh]) AND "Stroke/rehabilitation"[Mesh])) OR stroke) OR cerebrovascular accident) OR paralysis)) AND ((quality of life) OR "Quality of Life"[Mesh]))) AND ((tool* OR measure*))) AND Humans[Mesh]

**Mendeley search terms**

Quality of life tools for stroke, Stoke quality of life,
