# Supplementary material for: Post-stroke Quality of Life Index: A quality of life tool for stroke survivors from Sri Lanka
Source: Health Qual Life Outcomes. 2020 Jul 20;18:239. doi: 10.1186/s12955-020-01436-7 (PMC7370468; doi:10.1186/s12955-020-01436-7)
Supplement: Supplementary file 2 — Additional file 2. Domain analyses of commonly used QOL tools for stroke patients. [file 12955_2020_1436_MOESM2_ESM.docx]

**Supplementary file 2: Domain analyses of commonly used QOL tools for stroke patients**

| **Tool** | **Domains** |
| --- | --- |
| Cincinnati pre-hospital stroke scale ^64^ | Facial drop (1 item with two responses), arm drift (1 item with two responses), speech (1 item with two responses) |
| Stroke specific QOL scale ^10,65^ | Energy (3 items, each with five responses), family roles (3 items, each with five responses), language (5 items, each with five responses), mobility (6 items, each with five responses), mood (5 items, each with five responses), personality (3 items, each with five responses), self-care (5 items, each with five responses), social roles (5 items, each with five responses), thinking (3 items, each with five responses), upper extremity function (5 items, each with five responses), vision (3 items, each with five responses), work/ productivity (3 items, each with five responses) |
| Barthel index^66^ | Feeding (1 item with three responses), bathing (1 item with two responses), grooming, dressing (1 item with three responses), bowel habits (1 item with three responses), bladder functions(1 item with three responses), toilet use(1 item with three responses), transfers (1 item with four responses), mobility(1 item with four responses), using stairs (1 item with three responses) |
| SF-36 | General health (5 items with five responses), physical functioning (10 items with 3 responses), pain (1 item with six responses and 1 item with five responses), role-limitation-physical (4 items with two responses), role-limitation-emotional (3 items with two responses), social functioning (1 item with five responses and 1 item with four responses), fatigue (4 items with six responses), emotional well-being(5 items with six responses) |
| EQ-5D | Descriptive component (5 items; mobility, self-care, activity, pain, emotional well-being) and a VAS score (ranged from 0 to 100) |
| Scandinavian stroke scale | Consciousness (1 item with three responses), eye movement (1 item with three responses) , motor power; arm (1 item with five responses), motor power; hand (1 item with four responses), motor power; leg (1 item with five responses), orientation (1 item with four responses), speech (1 item with four responses), facial palsy (1 item with two responses) , gait (1 item with five responses) |
| London Handicap Scale (LHS) ^53^ | Mobility (1 item with six responses), orientation (1 item with six responses, work and leisure (1 item with six responses), social integration (1 item with six responses), physical independence (1 item with six responses), economic self-sufficiency (1 item with six responses) |
| Nottingham Health Profile (NHP) ^53^ | Part I: Physical abilities (8 items with two responses) , emotional reaction (9 items with two responses), social isolation (5 items with two responses), pain (8 items with two responses), energy level (3 items with two responses), sleep (5 items with two responses)  Part II: Life areas affected (7 items with two responses) |
| Sickness Impact Profile for Homes (SIP-NH) ^67^ | Ambulation (8 items with two responses), mobility(6 items with two responses), body care and movement (11 items with two responses), social interaction (9 items with two responses), emotional behavior (6 items with two responses), communication (5 items with two responses), alertness behavior (5 items with two responses), sleep and rest (4 items with two responses), eating (5 items with two responses), recreation and pastimes (7 items with two responses). |
| Frenchay activities index ^68^ | Domestic domain (5 items with four responses), Leisure/ work domain (5 items with four responses), Outdoors domain (5 items with four responses), |
